# Measurement of Quality of Life V. How to Use the SEQOL, QOL5, QOL1, and Other Global and Generic Questionnaires for Research

**DOI:** 10.1100/tsw.2003.80

**Published:** 2003-10-13

**Authors:** Soren Ventegodt, Joav Merrick, Niels Jorgen Andersen

**Affiliations:** The Quality of Life Research Center, Copenhagen K, Denmark

**Keywords:** Quality of Life, QOL, SEQOL, QOL5, QOL1, measurement, human development, holistic medicine, public health, Denmark

## Abstract

A survey can be divided into four phases: (1) planning, (2) distributing and collecting the questionnaires, (3) analysis, and (4) dissemination of the results. This paper provides step-by-step guidance on how the global and genetic SEQOL (self-evaluated quality of life) and QOL5 questionnaires can be used in a large- or small-scale survey of quality of life (QOL). It covers what is required to conduct a survey and generate results that should be of interest to local politicians, decision makers, and representatives from the surveyed groups and should be published after it has been processed and edited. If the results are to be published in the scientific literature, it is necessary to seek the assistance of an expert in statistics and/or research and the dissemination of research results.

## INTRODUCTION

This paper provides step-by-step guidance on how the global and genetic SEQOL (self-evaluated quality of life) and QOL5 questionnaires[1,2] can be used in large- or small-scale surveys. It covers what is required to conduct a survey and generate results that should be of interest to local politicians, decision makers, and representatives from the surveyed groups and should be published after it has been processed and edited. If the results are to be published in the scientific literature, it is necessary to seek the assistance of an expert in statistics and/or research and the dissemination of research results.

The quality-of-life (QOL) questionnaire measures two things: first, the dimensions of the quality of life, rated in eight different scales, leading to a global rating that is a weighted average of the subjective, existential, and objective dimensions. All ratings are expressed as a number between 10 and 90% (corresponding to quality-of-life ratings of "very poor" or "very good", respectively)[3].

Second, the questionnaire assesses various life domains (social factors, lifestyle, illness, sexuality, view of self, view of life, and values). These life domains can then be correlated with the quality of life, for instance, to determine whether people with a positive view of self have a higher quality of life or whether women with many induced abortions have a lower quality of life[4,5].

## THE GLOBAL, GENERIC QUALITY OF LIFE

Much medical research on the quality of life focuses on analyzing the effects and side effects of medicines; people are asked to describe how they feel after treatment. However, researchers have tended to concentrate on physical and mental side effects that are easily recognized. Side effects that are less tangible, such as general discomfort, slight depression, difficulty in concentration, insomnia, diminished communication skills, general burnout, and mental dissatisfaction receive less recognition in these studies. These side effects have been relegated to a category described as quality-of-life factors.

This approach makes it easy to tailor the quality-of-life concept to one's own research and goals, since it is not based on any sound theory. When the effect of pain relievers is examined, the quality of life might be defined as an indicator of how much of a headache one has. Advertisements in medical journals are based on this narrow definition of the quality of life. But this random, superficial definition of the quality of life, based on momentary commercial objectives, does not serve people's interests.

A good quality-of-life survey is therefore based on a broad, global concept of the quality of life, focusing on the total aspects of the life of the individual. We have tried to analyze all aspects of life, not just concentrating on factors relevant to one or several illnesses or treatments, such as cancer[6] or, more specifically, a benign enlargement of the prostate gland[7].

Quality-of-life rating scales based on the needs of one specific group, such as people with cancer or people in chronic pain, cannot be used in comparative studies of other groups. Quality-of-life rating scales specific to people with allergy cannot be used for people with heart problems and the results will be completely misleading if used on healthy people. These narrow rating scales cannot capture the quality of life of a major population group.

Nor can such rating methods based on specific health problems or treatments be used to compare the effect of different treatments on the same person. For example, a person who has leg pains might have a leg amputated. As there are different ways of measuring the quality of life of people in pain and people with limb loss, it is impossible to determine which treatment would provide the higher quality of life: is an amputation that relieves pain preferable to a life in pain but with both legs intact?

As one of the objectives of our survey was to compare various population groups (instead of factors of ill health), we have chosen generic factors that are theoretically common to people everywhere irrespective of age, sex, culture, and state of health. Measuring the quality of life presupposes that people can provide meaningful responses to the questionnaire. It should not be given to people with intellectual disability, but if the research is for this group it should instead be given to someone familiar with the person in order to give the responses, and we then have to assess whether such replies can be used meaningfully afterwards. Many people have questioned the concept of the quality of life with regard to treatment of these groups of people. Our findings show very clearly that decisions on treatment and priorities cannot merely rely on factors related to the quality of life; other factors have to be included as well.

People should realize that there are numerous quality-of-life questionnaires. They are mentioned in the scientific literature and can often be obtained from the researchers in question. You could develop your own questionnaire, but this is a complex process, albeit a valuable one, because you are then forced to be very explicit about what is meant by life and existence. Apart from the additional information that can be gained about the respondents, it gives a far better sense of what it means to carry out a questionnaire-based survey than is attained by simply using a ready-made questionnaire. However, it all boils down to resources: developing a new, small-scale questionnaire takes time — hundreds of hours of work.

A researcher may choose to combine a quality-of-life questionnaire — the complete SEQOL questionnaire[1], some of the eight component quality-of-life scales from SEQOL[8], just one quality-of-life scale from QOL5 or QOL1[2], or other quality-of-life scales — with a number of other questions that may seem particularly relevant or interesting to him or her. The rating scale for objective factors might be the most difficult to include. The researcher must be absolutely certain from the very start what is and is not to be rated in the survey. The Quality of Life Research Center in Copenhagen provides consultancy in preparing new questionnaires for specific aspirations, and the Center can also prepare such questionnaires.

Researchers may even choose to use only one quality-of-life question, the QOL1[2], to get a basic rating of the quality-of-life state. It is amazing how often hospitals neglect to compare how patients feel on admission with how they feel on discharge to ensure that patients believe that they have actually benefited from the treatment received. This can be assessed by simply asking patients about well being prior to admission and on discharge.

The rest of this paper assumes that the reader has chosen to use our questionnaire in a survey, although much of what is said below applies to all such surveys. A survey can be divided into four phases: (1) planning, (2) distributing and collecting the questionnaires, (3) analysis, and (4) dissemination of the results.

## Planning the Survey

### *Purpose*

Every survey must have a clear purpose[9]. Is the quality of life really the central theme of the survey, or might it be something else? Do not use a questionnaire simply because it is printed in a book that you have in front of you.

A survey may quickly occupy hundreds or thousands of working hours for the author and the respondents. Survey as few people as possible, and do not ask more than is needed for the project! Our questionnaire contains several subquestionnaires (each marked by a new title), and they can be used more or less independently.

Be certain what the purpose of the responses is. If the purpose is to improve the subjective well being of hospital patients, use the well-being rating scale and not objective factors. If the purpose is to rate how people with mental disorders function objectively, do not choose satisfaction with life, which rates the patient's subjective evaluation of life. Choose instead objective factors or realizing life potential, which are very objective.

The various rating scales are often mutually exclusive. Patients with therapeutically monitored days in the ward, human contact in a therapy group, and regular work in the therapeutic workshop may seem to function objectively well on the surface, yet may be deeply disillusioned with life and immensely frustrated because this contact is artificial and will cease once they are discharged. Nevertheless, these people may feel good in their everyday lives. If such a situation is only rated superficially, for example, the rating scales for well being and objective factors, the survey will miss the fact that everything is not rosy below the surface.

These factors suggest that the entire questionnaire should be used to ensure that global quality of life is rated. Be wary not to fall into the trap of only rating the respondents where the action is and failing to rate other areas where it is not, even if you feel that something ought to be going on.

Thus, if your purpose is narrowly defined, use quality-of-life rating scales suitable to this purpose. But it is generally wise not to define one's purpose too narrowly or to draw up a questionnaire too narrowly.

## *Identifying the Respondents*

It is generally clear whom one wishes to survey and why. You may be interested in a group who has been exposed to a certain intervention. But you may want to focus on a general set of problems and lack a group of people to survey. A telephone directory would be a fine source in such an instance: everybody on the third line from the bottom in the extreme left column of the directory could be phoned; after mentioning the purpose of the survey, you could ask for permission to send them a questionnaire.

A far more scientifically sound method would be to ask the Civil Registration System (CPR Register, Ministry of the Interior in Denmark) for help in selecting a random sample of people stratified by age, gender, or home address according to the distribution you want. The Civil Registration System in Denmark is happy to assist provided that you cooperate with a public authority who can guarantee data security, such as a municipality, university, or public hospital.

A small project might require only 50 respondents from one's own circle of acquaintances, colleagues, etc. In any case, a new questionnaire will require pilot tests on people you know, who are more likely to give their honest opinion about the questionnaire and the planned survey. Care must be taken not to strain people by giving them a questionnaire shortly after they have been given another questionnaire to fill out.

Start gradually; first family and friends, then colleagues and distant acquaintances, then people from the telephone directory (it might be a good idea to phone them first and ask whether they consent to filling out the questionnaire), and only draw on the Civil Registration System when the scientific standard of the survey requires it.

The quality of the accompanying letter or the explanation over the telephone are both crucial in the fate of the survey. It pays to think things over carefully in advance. A poorly planned survey must often be redesigned and then repeated, which is a nuisance to everybody, because the initial replies of the respondents have to be scrapped.

## *Resources*

If you are planning to undertake a questionnaire survey, like any other project in life, try not to bite off more than you can chew — a survey will probably take twice as long as you imagine initially.

It is a good idea to share the task of carrying out even a modest questionnaire survey with several colleagues. The members of your team should be given their tasks in advance, which will make everything so much easier in the long term. You must pay special attention to the less interesting tasks such as collecting data, editing, keying in, and writing articles. Who is to be responsible for all this? It is also a good idea to plan deadlines with which all members of the team will comply — hard work always requires some sort of discipline. Surveys of this type, no matter how exciting they are initially, invariably become hard work.

## Funding

A survey can be done in any price range and level of difficulty. A simple version is to take one question, such as "How do you feel now"? and give it to ten people on a piece of paper. This will give you an average quality-of-life score in 5 min and is easy to process manually.

A medium-sized survey comprises, say, 50 questions that might be put to two sets of 25 people. This will generate 2,500 responses and should be plenty of work for a term paper. Editing and keying in takes a couple of workdays. You would benefit from special software to cope with the task of keying in and the statistical work, although a spreadsheet capable of handling the most elementary analyses will do the job. If you want to move on and publish the results, it would be a good idea to seek professional help from a statistician. This might require at least 20 h at EURO 80/h, perhaps totaling EURO 5,000–10,000.

A major survey, comprising all eight quality-of-life rating scales, requires several hundred questions, perhaps even the entire questionnaire. A sensitive survey requires at least 20 people in each group, and you will soon reach a total of 100 respondents. This normally requires professional assistance and might require EURO 10,000 for statistical analysis. A survey of this size can easily occupy a part-time person for distribution, collection of data, guidance, etc. for 3–6 months. The Danish Quality of Life Survey at the Quality of Life Research Center had a total budget of about EURO 2 million (including the design).

You must therefore be quite sure how much money you need before you set out and apply for it, so that the money is obtained before the questionnaires are distributed.

## Approval and Protecting the Anonymity of the Respondents

If you want to survey patients, you must seek the prior approval of the committee on scientific ethics (each part of Denmark has such a committee) or sometimes called the Helsinki committee. This takes time, often several months. Information on the quality of life is classified as sensitive data, which means that all surveys (including those of nonpatients) processed electronically require the prior approval of the Danish Data Protection Agency.

Permission from the Danish Data Protection Agency is not required if the responses are anonymous (that is, the unnumbered questionnaire must state explicitly that the respondents do not have to provide their name). Protecting anonymity when the results of the survey are presented requires concealing certain information. For instance, if there was only one 88-year-old woman in a hospital ward, her responses must be omitted from the published table. This is another reason why even anonymous questionnaires must not be accessible to people not involved in the survey. They must be kept locked up when not in use and must be shredded once the survey is complete.

## Data Security

Data security is important; data must be encrypted, there must not be any modem link with the outside world, and a reliable system operator must ensure that passwords are taken seriously. Encryption means that the data are not accessible even if the data files or the entire computer were to be stolen.

All members of the survey team must sign an agreement in which they pledge never to take diskettes, questionnaires, or other material out of the office. Nevertheless, these security procedures do not protect against actual espionage carried out by a staff member. The person responsible for the project must set high ethical standards for the processing and protection of data. Each member of the research team must maintain these standards to ensure that there are no data leaks.

Security procedures in Denmark are agreed with the Danish Data Protection Agency. Every survey with an electronic registry should be presented to the Agency for security reasons, even if the survey is anonymous.

At the Quality of Life Research Center we take all necessary steps to ensure that a completed questionnaire — which often contains very sensitive information about deeply personal, intimate matters — never falls into the wrong hands. Unfortunately, a skilled private detective with the necessary time can identify the respondent to a questionnaire — even if it has not been marked with civil registration number, a name, or date of birth. If data end up in the wrong hands, anonymity can be broken by a person with the sufficient time, resources, and ill will.

Nevertheless, breaches in security, such as a questionnaire left on a desk for a few days, do not usually lead to any serious security threat, as very few people have both the necessary resources and ill will.

Computer files that contain the name, address, civil registration number, and similar information make it easy to identify the respondent. In contrast, the identity of the respondent cannot be traced using anonymous data files. In practice, however, most files lie somewhere in between these two types. It is therefore difficult to ascertain in practice exactly which data can make it easy to identify the respondent. These difficulties have contributed to the fact that both Danish and European Union legislation is not wholly adequate in this field.

In addition, data can vanish for various reasons, such as the breakdown of a hard disk. Frequent computer backup of data and results, ideally every day with a tape streamer, is mandatory. Computer viruses and theft are other reasons for data loss. A good, up-to-date antivirus program should be used regularly on the system, and computers should never start up with a disk in the disk drive. Every computer must be locked away and marked in some way, for instance, with VAT registration number or civil registration number etched into the frame, which make them less attractive to steal. Double locking systems, infrared sensors, and advanced alarm systems are necessary to prevent hackers and theft.

## Copyright and Ownership of Data

The old data a researcher wishes to build on in a survey may be protected by a copyright or other rights of ownership. If you receive a computer file from a person, who did not create the data himself or herself, you must seek the necessary permission to use it. The creator has the copyright. Yet files and disks can be owned by others than the person who created the data. In addition, institutions and other researchers may have permission to use the data as well.

Always get written permission to use the data, and if possible, get the data directly from the person who created it.

## Obtain Questionnaires

The Quality of Life Research Center will be happy to furnish individuals and groups with the questionnaire, especially for scientific, educational, and social welfare aspirations. We do not meet requests in which the advertising value of the quality-of-life concept is linked to specific products.

The SEQOL, QOL1, and QOL5 questionnaires may be freely copied for informal and scientific, noncommercial surveys; they may also be used for educational purposes. Permission for use in commercial research and nonscientific use must be obtained and the necessary number of copies of the original questionnaire must be ordered from the Quality of Life Research Center.

This procedure also ensures data security, and respondents can rest assured that their information will never fall into the wrong hands.

## Distribution and Collection of Data

### Distribution

The questionnaire must be distributed to all respondents at the same time, whether it is handed to them personally or mailed. In a nonanonymous survey, the name of the addressee should not appear on the questionnaire, as it might be stolen. Use a numerical code that can be identified using a central name index. Once the questionnaires have been received, they are renumbered to put them in order. Anonymous questionnaires are numbered after they are collected. Enclose a self-addressed envelope. Ensure that you receive the completed questionnaires and lock them up immediately.

### Informing the Respondents

The respondents must be thoroughly informed of the purpose of the survey and who is responsible for it. They must also be told that their data will be placed in a computer and that this information can be erased at any time if they want. Anyone who does not wish to be approached by researchers may write to the Civil Registration System in Denmark and request them not to give out his or her name and address for research purposes.

An accompanying letter must present the survey: who is responsible for it, its purpose, the deadline for returning the questionnaire, and to whom a respondent may direct a query. Both the Danish Data Protection Agency and the committees on scientific ethics have their own sets of rules on the information the respondents have to be given. These rules must be observed if your research is under the jurisdiction of the Agency or the system of ethics committees.

### Motivating the Respondents

The respondents must be well motivated to fill out and return the questionnaire. This will ensure a high response rate. It is an incentive in itself that somebody wants your response, and people in Denmark are generally very cooperative in scientific surveys, yet they may be critical of the value of them. You must therefore ensure that you get your message across and tell your respondents why it is so important that they invest their precious time in it.

The accompanying letter may encourage the respondents to return the questionnaire, but it must not pressure them to do so. Do not imply that their cooperation is part of a treatment they will not get if they do not respond. Ensure that they know that participation is completely voluntary and that, should they choose not to participate in the survey, nobody will know it.

The best way to motivate your respondents is to have a questionnaire that is interesting and with an appealing layout. This will entice them to just get started, and we have designed our questionnaire to be as informative and beneficial to the respondent as possible.

One way of motivating people, especially within the medical industry internationally, is to reward them with a small gift. Such a procedure will not work in Denmark because it gives the survey a commercial slant and that is not in accordance with research tradition in Denmark. So cultural aspects and attitudes have to be considered also.

Another way of getting responses is to tell your respondents that they will have access to the results. If only a few respond and the survey is not anonymous, they can simply be sent the results by mail. If there are many participants, it might be a good idea to inform respondents through professional journals or magazines. If the survey is huge, the mass media are likely to be interested and will publish selected results. However, since the mass media are unlikely to reach all the respondents, it is wise to promise as little as possible in terms of the mass media. It is important to keep all promises made, both because of common decency and because it ensures a positive perception of future surveys. All too often promises are made that are impossible to keep, because once the results are available, years later, all budgets have been exceeded and all resources exhausted.

### *Instructions*

A questionnaire must state in simple terms how the questions are to be answered and how the questionnaire is to be returned. If you want the response rate to be high, it might be a good idea to be available (for instance, on the telephone) as a number of potential respondents may misunderstand and misinterpret things and may not respond.

The respondents must be told to answer the questions in private and then put them in the self-addressed envelope and close the envelope. This will ensure honest, spontaneous responses.

### *Questionnaire Collection*

The return of two thirds of the questionnaires is a good response rate. Surveys of hospital patients often tend to generate 100% responses, as they feel obliged to participate. Postal surveys require hard work to obtain a response rate exceeding 50%[10].

Two weekends is a realistic deadline to give the respondents. This gives them enough time to fill in the questionnaire, but not enough time to mislay or forget it.

Do not expect that sending out reminders will have any major effect. This can be as costly as sending out the first batch of questionnaires. This is the case for anonymous surveys, where all respondents must be reminded, as well as for nonanonymous surveys, where those who have not responded can be identified. A reminder might increase the percentage of responses by 5 or 10%. Using reminders means that you have to ensure that subjects have not responded twice. The reminder must be diplomatic and not place unnecessary pressure on the respondent, and a questionnaire should be enclosed in case the original has been mislaid or deliberately thrown away.

Consider whether, instead of using a reminder, it might be better to prepare the initial batch at a much higher standard. Once again ensure that names, titles, and addresses are correct, spend a lot of time on the accompanying letter and the whole idea of the concept down to the color of paper and envelope, and give more and better guidance over the telephone.

Never send out a second reminder. A low response rate to the first reminder might indicate that you need to check everything for mistakes, wrong decisions, gaffes, etc. Instead of sending out a second reminder, try to find a different target group and do everything much better. The questionnaire that was used might have been too comprehensive, boring, or difficult to fill out and thus needs to be readapted.

## Processing and Analyzing the Questionnaire

This phase is difficult. The questionnaires have been returned and a lot of hard work lies ahead. It is easy to feel overwhelmed by the volume of material and to lose perspective.

The questionnaires must be numbered before they are processed. This is easiest with a stamp that automatically advances the digits from XX-0001, where XX is a year or other suitable code for the survey.

## Editing

Every questionnaire must be prepared so that it can be keyed in without too much trouble. Mistakes must be corrected, numbering codes for occupation and medicine must be checked, usually via computerized lists from the Danish Medical Association or Danmarks Statistik (the Statistical Bureau of Denmark).

Usually only 1% of the responses need editing. So this only poses a problem if the same question seems to be wrongly answered a number of times. However, editing takes just as much time as keying in.

## Data Entry

The Quality of Life Research Center supplies a dynamic data-entry program (QOL Analysis® in DOS and Windows) that can be adjusted to the current questionnaire. A fully electronic questionnaire that on completion produces the individual respondent's profile is also available (inquire for further details).

A spreadsheet might also be used in which each question is given a row, each respondent a column, and each response a cell. If you choose this solution, then you will need to do substantial work in planning how to key in each response.

A third possibility is to buy a keying-in program, for instance, a module for SAS® (a database system that can be extended with program modules). A small-scale survey can also be programmed to a pocket calculator.

Finally, you may send all your material to be processed elsewhere. Be aware that this may involve a security risk, as it can be difficult to guarantee the respondents the confidentiality of their responses when they are sent elsewhere. Ensure that the agency you choose for the job has good references, and remember that the least expensive agencies may be casual with security. Some agencies give very favorable price quotes that are exceeded in the final bill, since they charge extra for keying in small quantities of text.

## Scoring

When the data are edited and keyed in, the responses need to be scored — they must be converted to numbers in conformity with the various quality-of-life rating scales. Never use a questionnaire without familiarizing yourself with the scoring strategy. Surprisingly, several of the most popular rating scales for the quality of life have no scoring strategy, each researcher is expected to design one according to taste.

Scoring is always a problem when the rating scale is not based on a theory, which quality-of-life rating scales rarely are. The simpler the scales are, the simpler the scoring strategies will be, and the more they will gain in credibility and meaningfulness. Be very wary if the scoring strategy is similar to something derived from complex statistical procedures (for example, Rasch's item analysis); it may be based on pure statistics without considering reality and a good life.

The problem is that the more obscure the statistics are, the less clear the outcome of a survey will be. Statistical assistance is not always an advantage if it leads to methods for analysis that are difficult to interpret and use. It is not scientifically acceptable for a researcher to use methods he or she does not understand. Therefore, always choose rating scales that are thoroughly reasonable, including questions, responses, theoretical background, and scoring strategy. Do not use a rating scale unless every detail related to editing and data processing is settled.

## Weighting

The ideal thing is to establish the weighting as part of one's theory. However, the objective quality of life is very random and culturally conditioned, and these facts determine how a list is put together. We must all feel free to seek good reasons to weight the questions differently. So use or change them so that they fit your cultural norms.

## Analysis

The data must be analyzed in accordance with the purpose of the survey. At this point, hypotheses are made regarding relationships, and parts thereof, to make them testable. It is too easy to rationalize by simply aimlessly surfing for data. Also, remember the problem of statistical significance: a level of 0.05 means that every twentieth statistically significant result will reflect random error and not indicate a true correlation.

We recommend that you use a broad set of quality-of-life rating scales and that you use our three-dimensional landscapes, which make the results immediately comparable to ours.

If the purpose of a survey is to compare two groups of respondents, such as people before and after treatment (X) or after treatment (Y) or treatment (Z), respectively, then you must remember to look at the average ratings of quality of life of the two groups. We consider a difference of 40% to be very large, corresponding to two steps on a five-step scale, for instance, from "poor" (30%) to "good" (70%). We consider a difference of 20%, corresponding to one step, to be large, 10% to be moderate, 5% to be small, and less than 5% to be very small. Large differences are rare and small differences common.

If you wish to know whether the difference is significant (at $p = 0.05$, that is, whether it would occur due to random error in less than 1 of 20 cases), you can look at our tables and see whether the number of respondents used lets you draw this conclusion (provided that the variance is not significantly different from the one attained in our survey). If the number of respondents differs from that given in our survey, you may interpolate from the three pairs of numbers by means of logarithmic paper and a ruler, using millimeter paper and a freehand drawing, or you may work out the exact numbers with the necessary statistical guidance provided.

Correlation or regression may be used if you wish to explore the relationship between one life domain (one factor) and the quality of life.

You can work out the correlation between pairs of observations on a pocket calculator or any statistical program. A number of correlations are typically between 0.1 and 0.3, which expresses a link between more remotely related factors; 0.5 shows a strong link worth investigating that cannot be said to be trivial in theoretical terms. Links above 0.7 often reveal that the same factor is being measured in more than one way or instance.

We recommend that you use linear regression if you want to determine the difference in the quality of life between those with the lowest score in the factor investigated (for instance, personal income) and those with the highest score. The regression will then calculate a minimum and a maximum above the measured interval, and the numbers stated above are also valid here: a difference of more than 40% between minimum and maximum is very large.

### *Presenting the Results*

A computer will enable you to present your data efficiently and attractively. Only zealous research colleagues will be interested in the numbers in tables. Use the potential of spreadsheets to achieve the best possible graphic design, as we have done. The snag is, however, that most people lose their intuitive feel of what a three-dimensional graph means. It does take some concentration to learn to read such a graph, but once you have the hang of it, you will never wish to see a two-dimensional graph again: it is far more meaningful to study three-dimensional graphs, because of the wealth of information they generate.

### Dissemination

There are two obvious targets for the disseminated results of a survey: those who must know the outcome for the aim of the survey to be fulfilled (notably colleagues or political and administrative decision makers) and the patient or population group under investigation (for instance, patient associations, speakers on the topic, or opinion leaders).

For the first group, publication in the usual professional or scholarly magazines or journals is appropriate. As to the second group, consider how to notify the respondents to thank them for their cooperation. If your survey is large and generates interesting results, you may consider notifying the press and the electronic media. A one-page press release faxed to 20 newspapers and television and radio stations, especially the local ones, is easy to accomplish and may be very valuable.

## CONCLUSION

A quality-of-life difference might be statistically significant, without being of practical significance. Another use of global quality-of-life rating scales, that makes it so valuable, is that an intervention can measure the quality-of-life gain, which has practical significance for evaluation. We used to call an absolute difference of 5% a small, but practically significant, difference. This seems to be the case for all questionnaires developed with the method described elsewhere[3], based on a distinct quality-of-life theory[11] and using the optimal combination scale[12].

It is helpful for further investigations if national data are available making it possible to discriminate between high and low quality of life. SEQOL is easy to use in Denmark and the Nordic countries now, as there exist results for the Danish population, but we hope soon to be able to present data for other countries as well. We also hope that researchers carrying out research on the global quality of life will use national samples whenever possible, to contribute to the knowledge of the quality of life of our world, facilitating the research worldwide.

We have demonstrated how to make a scientific quality-of-life survey, but the most critical part is selecting the right questionnaire(s). It is of crucial importance that the collected information is of sufficient quality for the purpose of the study. The investigated dimensions must be exactly the dimensions that need examination. A quality-of-life survey will never be better than the selected questionnaire. We recommend for most general purposes the use of a global and generic measure, like SEQOL, QOL5, or QOL1, as the meanings of the collected data are easy to interpret, compared to standard questionnaires used in medical surveys, like the often-used health-related Sickness Impact Profile and the Nottingham Health Profile. Of course, if there is a tradition in the field of research, it is wise (also) to use the standard tools, not to miss the opportunity to compare the results. When the appropriate questionnaire(s) are found, the steps outlined here, when carefully followed, will secure that the quality-of-life survey is running smoothly and without unexpected trouble.

## ACKNOWLEDGMENTS

This study was supported by grants from The 1991 Pharmacy Foundation, as well as by supplementary grants from Goodwill-fonden, the JL-Foundation, E. Danielsen and Wife's Foundation, Emmerick Meyer's Trust, the Frimodt-Heineken Foundation, the Hede Nielsen Family Foundation, Petrus Andersens Fond, Wholesaler C.P. Frederiksens Study Trust, Else & Mogens Wedell-Wedellsborg's Foundation, and IMK Almene Fond. We gratefully acknowledge the critical scrutiny and expert linguistic assistance of Ib Ravn, Ph.D. The research was approved by the Copenhagen Scientific Ethical Committee under number (KF)V.100.2123/91.

## References

1. Ventegodt, S., Henneberg, E.W., Merrick, J., and Lindholt, J.S. (2003) Validation of two global and generic quality of life questionnaires for population screening: SCREENQOL & SEQOL. *TheScientificWorldJOURNAL* **3**, 412–421.
2. Lindholt, J.S., Ventegodt, S., and Henneberg, E.W. (2002) Development and validation of QOL5 for clinical databases. A short, global and generic questionnaire based on an integrated theory of life. *Eur. J. Surg.* **168,** 107–113.
3. Ventegodt, S., Hilden, J., and Merrick, J. (2003) Measurement of quality of life I. A methodological framework. *TheScientificWorldJOURNAL* **3**, 950–961.
4. Ventegodt, S. (1995) *Quality of Life in Denmark. Results from a Population Survey*. Forskningscentrets Forlag, Copenhagen.
5. Ventegodt, S. (1996) *The Quality of Life of 4500 31-33 Year-Olds. Result from a Study of the Prospective Pediatric Cohort of Persons Born at the University Hospital in Copenhagen.* Forskningscentrets Forlag, Copenhagen.
6. Bjordal, K. and Kaasa, S. (1992) Psychometric validation of the EORTC core quality of life questionnaire, 30 item version and a diagnosis-specific module for head and neck cancer patients. *Acta Oncol.* **31(3),** 311–321.
7. Epstein, R.S., Deverka, P.A., Chute, C.G., Panser, L., Oesterling, J.E., Lieber, M.M., Schwartz, S., and Patrick, D. (1992) Validation of a new Quality of Life Questionnaire for benign prostatic hyperplasia. *J. Clin. Epidemiol.* **45(12),** 1431–1445.
8. Ventegodt, S. (1996) *Measuring the Quality of Life. From Theory to Practice*. Forskningscentrets Forlag, Copenhagen.
9. Cella, D.F. and Tulsky, D.S. (1993) Quality of life in cancer: definition, purpose and method of measurement. *Cancer Invest.* **11(3),** 327–336.
10. Brorson, B., Ifver, J., and Hays, R.D. (1993) The Swedish health related quality of life survey (SWED-QUAL). *Qual. Life Res.* **2,** 33–45.
11. Ventegodt, S., Merrick, J., and Andersen, N.J. (2003) Quality of life theory I. The IQOL theory: an integrative theory of the global quality of life concept. *TheScientificWorldJOURNAL* **3**, 1030–1040.
12. Ventegodt, S., Merrick, J., and Andersen, N.J. (2003) Measurement of quality of life II. From the philosophy of life to science. *TheScientificWorldJOURNAL* **3**, 962–971.

## BIOSKETCHES

**Søren Ventegodt, MD,** is the Director of the Quality of Life Research Center in Copenhagen, Denmark. He is also responsible for a Research Clinic for Holistic Medicine in Copenhagen and is a popular speaker throughout Scandinavia. He has published numerous scientific or popular articles and a number of books on holistic medicine, quality of life, and quality of working life. His most important scientific contributions are the comprehensive SEQOL questionnaire, the very short QOL5 questionnaire, the integrated quality-of-life (IQOL) theory, the holistic process theory, the life mission theory, and the Danish Quality of Life Research Survey, 1991–94 in cooperation with the University Hospital of Copenhagen and the late pediatric professor Bengt Zachau-Christiansen. E-mail: ventegodt@livskvalitet.org. Website: www.livskvalitet.org/

**Joav Merrick, MD, DMSc,** is Professor of Child Health and Human Development affiliated with the Zusman Child Development Center and Division of Community Health at the Ben Gurion University, Beer-Sheva, Israel and presently the Medical Director of the Division for Mental Retardation, Ministry of Social Affairs, Jerusalem and the Director of the National Institute of Child Health and Human Development. He has numerous publications in the field of child and human development, rehabilitation, intellectual disability, disability, health, welfare, abuse, advocacy and prevention. Dr. Merrick received the Peter Sabroe Child Award for outstanding work on behalf of Danish Children in 1985 and the International LEGO-Prize ("The Children's Nobel Prize") for an extraordinary contribution towards improvement in child welfare and well being in 1987. E-mail: jmerrick@internet-zahav.net. Website: www.nichd-israel.com

**Niels Jørgen Andersen, MSc,** Professor, Department of Innovation and Economic Organization, Norwegian School of Management. This department conducts research and provides teaching in central topics related to innovation, business development, management of global companies, business history, and economic organization. Research activities within the Department are related to four core subjects within the discipline: business history, cooperative organizations, business development and entrepreneurship, and finally studies of industries with a special focus on the electricity industry. He is also the dynamic chairman of the nonprofit organization Stiftelsen Holistisk Medisin Scandinavia, which aims to support the scientific development, research, and documentation of complementary and holistic medicine in Scandinavia. E-mail: niels.j.andersen@bi.no. Website: www.bi.no/users/fgl93013/
